# Influence of Ultrasound and the Conditions of Convective Drying with Dehumidified Air on the Course of the Process and Selected Properties of Apple Tissue

**DOI:** 10.3390/foods10081840

**Published:** 2021-08-09

**Authors:** Aleksandra Matys, Artur Wiktor, Magdalena Dadan, Dorota Witrowa-Rajchert

**Affiliations:** Department of Food Engineering and Process Management, Institute of Food Sciences, Warsaw University of Life Sciences—SGGW, 02-787 Warsaw, Poland; aleksandra_matys@sggw.edu.pl (A.M.); magdalena_dadan@sggw.edu.pl (M.D.); dorota_witrowa_rajchert@sggw.edu.pl (D.W.-R.)

**Keywords:** apple, ultrasound, convective drying, dehumidified air, quality

## Abstract

The conditions of convective drying induce a negative effect on the quality of the dried products, and thus, innovative solutions, such as the utilization of ultrasound and dehumidified air are gaining importance. Therefore, the aim of this study was to evaluate the impact of ultrasound pretreatment and variable air temperature on the kinetics of convective drying with dehumidified air and the quality of dried apples. Apples were dried with dehumidified and non-dehumidified air at 55, 70 and 85 °C. Preliminary ultrasound treatment was conducted by immersion for 60 min. The effect of both ultrasound and dehumidified air utilization was more prominent in the terms of drying time reduction, when lower drying temperatures were used. Drying of apples with dehumidified air, preceded by ultrasound pretreatment, resulted in a greater rehydration capacity of the products, and limited the browning process. Dehumidified air increased the lightness of the dried products, while sonication darkened them. The use of ultrasound before drying coupled with a drying with dehumidified medium at a temperature of 70 °C reduced the exposure of the product to a high temperature and oxygen. Products treated before dehumidified air drying with ultrasounds were characterized by high total phenolic content and the greatest antioxidant activity. This was because such technological treatment reduced exposure of the product to a high temperature and oxygen.

## 1. Introduction

Convective drying (hot air drying) is traditional method used frequently for dehydration of different food materials [1] by hot air. Hot air supplies heat to the material and removes moisture from it. Less frequently, an inert gas or superheated steam are used instead of it [2,3,4]. More than 90% of dried food is obtained by convective drying [5]. This method consumes over 25% of the energy used by the industry in developed countries [6,7]. Hot air dryers are characterized by simple construction [8], relatively low costs, and easy control. Among the disadvantages, a significant energy consumption [9,10], a low thermal efficiency, and long drying times can be mentioned, resulting in an unsatisfactory quality of the obtained products [1]. The main reason of such situation is a large amount of unused thermal energy, a low thermal conductivity of the product, and the significant latent heat of water vaporization [8]. The long exposure of the product to a high temperature and oxygen leads to numerous adverse physical changes in the product [2]. It induces a destructive effect on the labile bioactive compounds, thus, significantly reducing nutritional and sensory quality of dried material [11]. The proper selection of the process parameters makes it possible to limit the negative impact of the convective drying on the quality of the dried products. An interesting solution is modification of a convective dryer to combine it with an air dehumidifier.

It is worth emphasizing that the drying kinetics is related to the moisture content in the drying air. Although performing drying process at ambient humidity is advantageous due to the simplicity of the experimental setup, one of the significant problems is the unrepeatability of the process. Indeed, the humidity of the drying medium depends on the changes of ambient conditions. A reduction of the amount of water in the air increases the potential of the drying medium to absorb moisture, i.e., to remove it from the product. This is due to the increased difference in water vapor pressure between the product’s surface and the air, causing an increase in the driving force of mass transfer during drying [12,13]. Dehumidified air is most often used in spray drying [14] and fluidized bed drying [12] systems. Whereas, the application of dehumidified air in the hot air drying has not been well analyzed. Kaya et al. [15] studied the effect of the parameters of drying air, such as temperature, velocity and relative humidity, during drying of kiwifruit. The results revealed that a decrease in the relative air humidity from 85 to 40% (at a temperature of 35 °C and the air velocity of 0.3 m/s) led to a reduction of drying time by 56%.

The intact cell membrane of the plant tissue constitutes a sort of barrier that limits diffusion. Therefore, scientists are looking for solutions that, by interfering with the cell membrane, may improve the drying process and ensure the appropriate quality of the dried product [16]. One of these solutions is the use of an ultrasound. The ultrasonic waves, penetrating throughout the product, cause a sequence of repeatable compressions and decompressions therein. This phenomenon is called the “sponge effect” because the product resembles a compressed sponge that is then decompressed [8,10]. The forces generated during this process may displace water from the inside of the product’s tissue toward its surface, and lead fluids into the product from the outside [1,17]. Acoustic waves cause the formation of microchannels between cell clusters in the product’s structure, through which the intracellular fluid may leak into the surrounding medium [10]. The changes in a product subjected to high-intensity ultrasound result from the acoustic cavitation, which arises from the interaction between ultrasonic waves, a liquid medium, and a gas [18,19]. Cavitation is the formation, enlargement, and/or collapse of gas bubbles in a liquid medium, contributing to the sudden evaporation of moisture and mechanical injury of the treated material, depending on the type of cavitation. Ultrasound can led to inertial and non-inertial cavitation. In the case of inertial cavitation the bubbles collapse and can rupture the cell. Accordingly, non-inertial cavitation ultrasound bubbles have longer lifetimes and can contribute to the morphological changes in the structure using the “sponge effect”. It is believed that both types of ultrasound induced cavitation can cause perforation of the cell membrane, but inertial cavitation can do it faster [20]. Ultrasonic energy may widen the intercellular spaces and capillary microchannels in the structure of the tissue, thus, intensifying the internal transport of water [1]. The influence of the ultrasound was reported both as the release of water strongly bound in the structure of the product [9], and as the removal of oxygen dissolved in the intercellular spaces of tissues (degassing) [21]. The occurrence of the phenomena mentioned above creates the possibility of improving water diffusion, which should basically increase the rate of water removal from the product. The application of ultrasound prior to water removal from food materials has been reported before in some publications. One of the first publications about utilization of ultrasound in this field concerned osmotic dehydration of apple cubes [22]. The authors demonstrated that sonication intensifies mass transfer and influences both solid gain and water loss flux. The first articles which dealt with the ultrasound applied before drying the concerned banana or button mushrooms [23,24]. Recently, ultrasound pretreatment has been demonstrated for kiwi, carrot, and garlic drying [21,25,26]. Most of the research published in this field refers to hot air drying, but there are some examples where other drying methods were studied–for instance, infrared drying [27] or microwave drying [28]. However, until now, the combination of ultrasound with dehumidified air drying, and its impact on process kinetics and product quality has not been tested before.

The aim of this study was to determine the kinetics of the ultrasound-pretreated apples dried by the means of convective drying with dehumidified air carried out at variable air temperature, as well as to compare selected quality properties of the obtained products.

## 2. Materials and Methods

### 2.1. Material

The material used in this study included ‘*Golden Delicious*’ apples obtained from the Experimental Fields of the Department of Fruit Growing of the Warsaw University of Life Sciences (SGGW, Poland). Prior to the experiment, the fruits were stored in the dark at the temperature of 4–5 °C in a relative air humidity of 80–90% for one week. Subsequently, the apples were cut into 5 mm-thick slices and stripped of cores. The slices were cut into four equal pieces. The product was dried immediately with non-dehumidified and dehumidified air or subjected to an ultrasound pretreatment and then dried with dehumidified air.

### 2.2. Ultrasound Pretreatment

The sonication of the apples (131.6 ± 1.3 g) was carried out by immersion in an ultrasonic bath (MKD-3, MKD Ultrasonics, Stary Konik, Poland). The pretreatment was conducted in a continuous mode, with the ultrasound frequency of 21 kHz and the power of 180 W for 60 min. The parameters of sonication were adjusted experimentally (data not shown). The ratio of raw product mass to water mass was 1:4. The product, together with water, was placed in a glass beaker and covered with a Petri dish so as to completely immerse material in water. The beaker was placed in the ultrasonic bath filled with distilled water. The temperature of the water for sonication was 21.7 ± 1.6 °C and, after the process, it increased up to 22.8 ± 2.0 °C. After the ultrasound pretreatment, excess water was removed from the product with filter paper and the product was, then, dried convectively with dehumidified air. After sonication, the mass of apples increased by 5.2 ± 3.0 g.

### 2.3. Convective Drying

**Figure 1:**
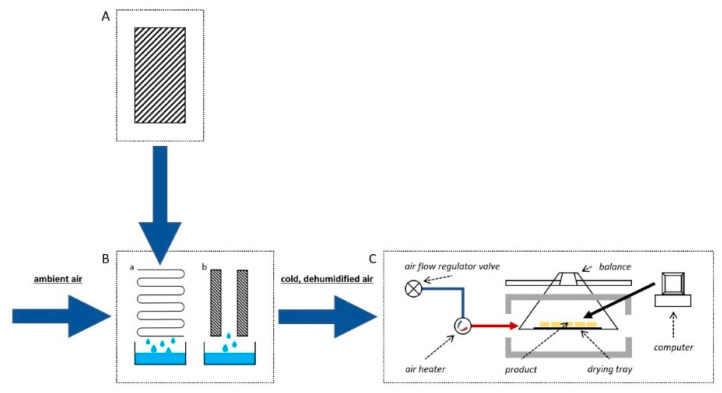
Scheme of drying equipment used in the study. (**A**)—cooling unit; (**B**)—dehumidification system which consist of a—condensation and b—adsorption parts; (**C**)—dryer.

The apples were dried in a laboratory convective dryer, which was additionally equipped with an air dehumidification system (Figure 1). The dehumidifier consisted of a cooling unit (MTA, TAEevo TECH020, MTA, Tribano, Italy) and a condensation-adsorption unit (ML270, Munters, Sweden). Air humidity after dehumidification was equaled to 1.5 g/m^3^. The air (temperature: 55, 70, 85 °C; velocity: 1.5 m/s) flowed parallel to the layer of the product placed on the sieve. The load on the sieve was 1.25 kg/m^2^. Every five minutes, changes in the mass of the apples during drying were recorded, with an accuracy of ±0.1 g. The samples were dried to a constant mass. The process was performed in duplicate.

Based on the records of changes in the mass of the product during drying, the drying curves were plotted, being a relationship between the relative (dimensionless) water content and the time, *MR* = f(τ):

(1) $$MR=\frac{M_{\tau }}{M_{0}},$$

where *M_τ_* is the water content during drying [kg H_2_O/kg d.m.] and *M*_0_ is the initial water content [kg H_2_O/kg d.m.].

The drying time was defined as the time necessary to obtain *MR* = 0.02.

### 2.4. Dry Matter Content

The analysis of dry matter content in fresh and dried material was performed with the gravimetric method. The samples were dried in a vacuum laboratory dryer at 70 °C for 24 h without air circulation until constant mass was achieved according to AOAC 920.15, 2002 standard [29]. The measurement was performed in duplicate for each obtained material.

### 2.5. Water Activity

The measurement of water activity in fresh and dried material was performed with a hygrometer (AquaLab CX-2, Decagon Devices, Pullman, WA, USA) at room temperature (approx. 25 °C) in duplicate for each obtained material.

### 2.6. Hygroscopic Properties

The water vapor adsorption capacity (*m_τ_*/*m*_0_) of the dried product was analyzed according to a method proposed by Wiktor et al. 2019 [30]. A material of a known mass was placed in a desiccator over a saturated NaCl solution, whose water activity was 0.75. The measurement was performed at room temperature (approx. 25 °C). The samples were reweighed after 30 min, and 1, 2, 3, 6, 9, 24, 48, and 72 h. The results were expressed as the change in the ratio between the mass of the product during the measurement (*m_τ_*) and its initial mass (*m*_0_). The measurement was performed in duplicate for each obtained material.

### 2.7. Rehydration Properties

The rehydration properties of the dried apples were analyzed with a modified method proposed by Witrowa-Rajchert and Lewicki [31]. One piece of a dried apple was placed in 80 cm^3^ beaker and 40 cm^3^ of distilled water at a temperature of approximately 20 °C was added. After 30 minutes, the material was separated from the water; excess water was removed with filter paper and the apple was reweighed. After comminution, in all studied materials the dry matter content was determined. The analysis was done in duplicate. Based on the results, the relative mass gain (*Δm*) and the relative dry matter cotent (SSL) were calculated:

(2) $$\Delta m=\frac{m_{\tau }}{m_{0}},$$

(3) $$SSL=\frac{m_{\tau }\times dm_{\tau }}{m_{0}\times dm_{0}},$$

where *m_τ_* is the mass of the sample after rehydration [g], *m*_0_ is the mass of the sample before rehydration [g], *dm_τ_* is the dry matter content in the sample after rehydration [%], and *dm*_0_ is the dry matter content in the sample before rehydration [%].

### 2.8. Color

The color of a fresh and a dried product was determined with a trichromatic colorimeter (CR-5, Konica-Minolta, Japan). The analysis was performed with the reflected light and the CIE L*a*b* system (light source: D65; standard observer: 2°; diameter: 8 mm). The measurement was performed in ten replications for each obtained material. Based on the recorded values of the L*, a*, and b* color parameters, the total color difference (*ΔE*) and the browning index (*BI*) were calculated as follows:

(4) $$\Delta E=\sqrt{{\left(\Delta L^{*}\right)}^{2}+{\left(\Delta a^{*}\right)}^{2}+{\left(\Delta b^{*}\right)}^{2}},$$

(5) $$BI=\left[\frac{100\times \left(\left(\frac{a^{*}+1.75L^{*}}{5.645L^{*}+a^{*}-0.3012b^{*}}\right)-0.31\right)}{0.17}\right],$$

where ΔL*, Δa*, Δb*—the differences between the values of the L*, a*, b* of dried and fresh apples.

### 2.9. Total Phenolic Content

The analysis of the total phenolic content in fresh and dried apples was performed according to the Folin-Ciocalteu’s method [32] with gallic acid as a standard. The method was previously described in detail [33]. A spectrophotometer (Heλios Thermo Electron v. 7.03, Thermo Electron Corporation, Waltham, MA, USA) was used to measure the absorbance of the extracts at a wavelength of 750 nm against blank sample. The extracts was prepared in duplicate. The total polyphenol content in the sample was expressed in milligrams of gallic acid per 100 g of dry matter (d.m.).

### 2.10. Antioxidant Activity

Antioxidant activity was determined based on the degree of scavenging of DPPH•-that is, 2,2-diphenyl-1-picrylhydrazyl synthetic radical-by antioxidants extracted from the samples. The measurement of absorbance at a wavelength of 515 nm was made with a spectrophotometer (Heλios Thermo Electron v. 7.03, Thermo Electron Corporation, Waltham, MA, USA). The analysis was repeated twice. The antioxidant activity was expressed as the EC_50_ coefficient, defining the concentration of the extract necessary to reduce the initial amount of DPPH• radicals by 50% [34,35].

### 2.11. Statistical Analysis

One-way analysis of variance ANOVA, was performed with the statistical software Statistica 13.3 (TIBCO Software, Palo Alto, CA, USA). The results were assigned to homogeneous groups using Tukey’s test (α = 0.05).

## 3. Results and Discussion

### 3.1. Drying Kinetics and Drying Times

**Figure 2:**
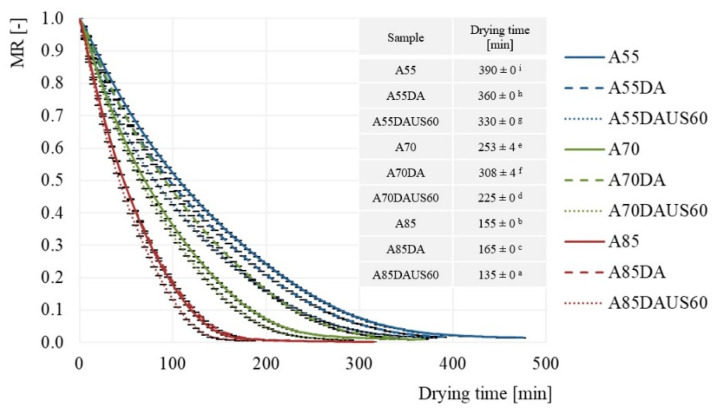
Drying kinetics and drying times of apples using the convective method with non-dehumidified air, dehumidified air, and dehumidified air with ultrasound pretreatment; the same letters (a, b, etc.) in the columns represent homogeneous groups in statistical terms (α = 0.05).

The drying curves and the times required for the dried apples to reach MR = 0.02 are shown in Figure 2. Based on the course of the curves, it may be concluded that the variable temperature and humidity of the drying air, as well as the applied ultrasound pretreatment influenced the kinetics of the convective drying process (*p* < 0.05). Notably, an increasing of the air temperature resulted in a reduction of drying time. The apples were dried with non-dehumidified air at 55, 70 and 85 °C for 390, 253, and 155 min, respectively. In comparison to the same temperature of ambient air, the application of dehumidified air reduced the drying time by 30 min when 55 °C was set. Whereas, for 70 and 85 °C the drying time was longer by 55 and 10 min, respectively. The benefits of using dehumidified air of relatively low temperature in convective drying were described in the literature. Kaya et al. analyzed the impact of temperature (35−55 °C), velocity (0.2–0.6 m/s), and relative air humidity (40−70%) on the convective drying of apple [36] and quince [37]. Reduction of the relative humidity from 70 to 40% increased the drying rate and reduced the process by 52% for apple and by 64% for quince (at the temperature of 35 °C and the air velocity of 0.2 m/s). In both cases, the reason could have been the increase in the difference in water vapor pressure between the surface of the dried product and the drying air, improving the mass transfer process. Ultrasonic waves reduced the drying time of apples in each of the set values of the drying air temperature, but their influence was the greatest at the lowest temperature. When the air temperature was 55 °C, the application of ultrasound treatment prior to drying with dehumidified air resulted in a reduction of the drying time by 60 min in comparison to drying of apples not subjected to the ultrasound and conducted with the use of non-dehumidified air. Numerous authors [16,38,39,40,41,42] have reported a reduction of the drying time after the application of ultrasound pretreatment, which facilitates water loss by accelerating its diffusion during convective drying. At lower temperatures of the drying process, the influence of ultrasonic energy is significant, but decreases with the increasing temperature of the drying air [43]. The reason why ultrasound enhances drying kinetics is associated with the changes that it provokes at the cellular level. As it was reported previously in the literature it creates microchannels in the structure and causes cell disintegration at the tissue level, especially on the surface of the product, improving the diffusion processes [3,16,38,44,45,46].

### 3.2. Dry Matter Content and Water Activity

Table 1 shows the average dry matter content in the dried apples, which varied from 91.09 to 98.88%. As the temperature of the drying air rose, the amount of water removed from the product increased. Dehumidification of the air at each of the set temperatures led to obtain dried product with a lower dry matter content than in the case of drying with non-dehumidified air. The apples subjected to ultrasound pretreatment followed by drying with dehumidified air at a temperature of 55, 70, and 85 °C were characterized by respectively lower, higher, and statistically identical dry matter content as the products obtained with the same drying conditions, but untreated by ultrasound (*p* < 0.05). The dry matter content in the dried apples was consistent with the literature data [38,40,41].

Table 1 shows also the water activity of the dried apples, with all products reaching values in the range of 0.195–0.339. At the same time, the obtained products had a water content in the range of 1.12–8.91%, which indicates their stability. The increase in temperature during drying contributed to a decrease in the water activity of the obtained products. The use of dehumidified air at the temperature of 55 and 70 °C increased the water activity of the products in comparison to non-dehumidified air, but with the temperature increasing to 85 °C, the water activity of the product dried with dehumidified air and did not differ statistically from apples dried with non-dehumidified air (*p* > 0.05). Ultrasound treatment prior to drying with dehumidified air at 55 and 85 °C resulted in products with the highest water activity among the apples dried at these temperatures. However, in the case of a drying air temperature of 70 °C, pretreatment with ultrasonic waves produced dried apples with the lowest water activity among products dried at this temperature. In this case, water activity was equal to 0.223, which means that it was lower by 4.3 and 23.2% when compared to sample dried with ambient and dehumidified air, respectively. These differences in water activity levels for untreated and pretreated materials dried at different temperatures may suggest that presence of pretreatment can lead to some changes in the state of water or crystallinity, as it was reported previously for pulsed electric field treated freeze-dried apples, due to disintegrated structure of the material [47]. However, this theory needs further investigation to be confirmed. The higher dry matter content in the dried apples, the lower was their water activity. Similar results of the water activity of dried apples were reported in the literature previously [38,40,41].

### 3.3. Hygroscopic Properties

**Figure 3:**
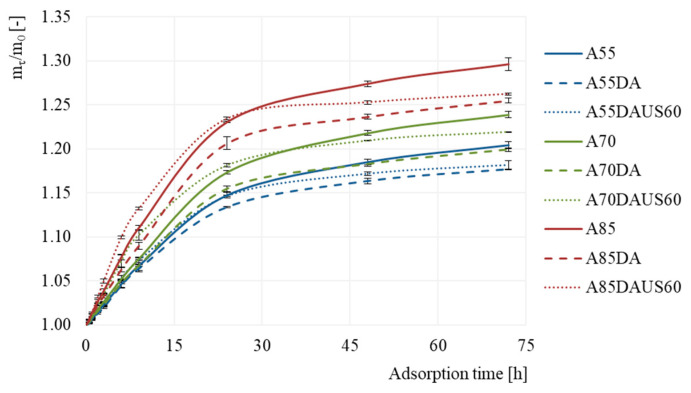
Mass gain depending on the time of moisture adsorption by the dried apples obtained by the convective method with non-dehumidified air dehumidified air, and dehumidified air with ultrasound pretreatment.

**Figure 4:**
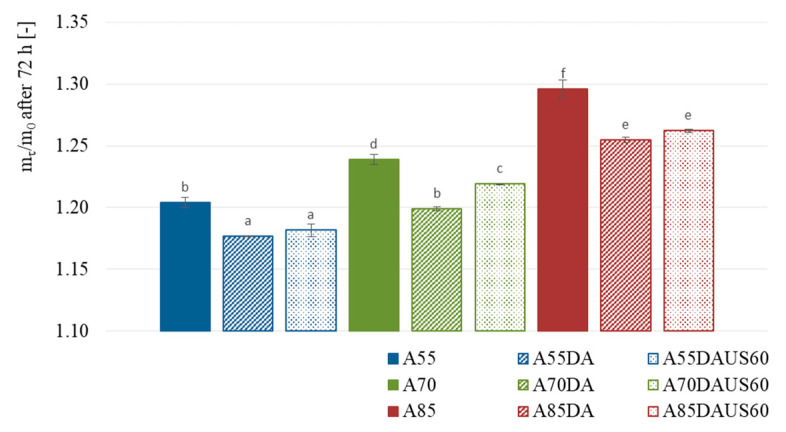
Mass gain after 72 h of moisture adsorption by the dried apples obtained by the convective method with non-dehumidified air, dehumidified air, and dehumidified air with ultrasound pretreatment; the same letters (a, b, etc.) in the columns represent homogeneous groups in statistical terms (α = 0.05).

Figure 3 shows the mass gain of the dried apples versus time of moisture adsorption. The ability of the samples to adsorb water vapor decreased with time. The products of drying with non-dehumidified air of 85 °C more intensively adsorbed water vapor. Whereas, the lowest mass gain was obtained in the case of apple dried with dehumidified air of 55 °C. It seems that the dry matter content in dried apple (Table 1) was crucial factor differentiating the ability of dried product to adsorb the moisture from the environment. Figure 4 presents the mass gain of the dried apples after 72 h of determination. The higher the air temperature, the higher was the mass gain, irrespectively of the other processing conditions (*p* < 0.05). In each of the set values of the air temperature (55, 70 and 85 °C), the apples dried with non-dehumidified air were characterized by a greater mass gain than those dried with dehumidified air. Moreover, the highest hygroscopicity was noted in the case of A85 sample, which may indicate less damage of the apple tissue caused after this drying. Dehumidification of the air at each of the set temperatures led to obtain dried apples with the lowest hygroscopic properties. When both the lowest and the highest air temperature were set, the application of ultrasound pretreatment resulted in a similar hygroscopicity as in the case of apples dried with dehumidified air. Only in the case of 70 °C, the sonication led to a better water vapor adsorption. Dadan and Nowacka [48] reported both increased and decreased hygroscopicity after sonication, depending on the time of application. Authors explained results by the changes in the internal structure and possible collapse during drying. In turn, Fijałkowska et al. [41] observed worse hygroscopicity after applying ultrasound treatment prior to convective drying of apples. This could be induced by changes in the structure of biopolymers responsible for binding water [49].

### 3.4. Rehydration Properties

**Figure 5:**
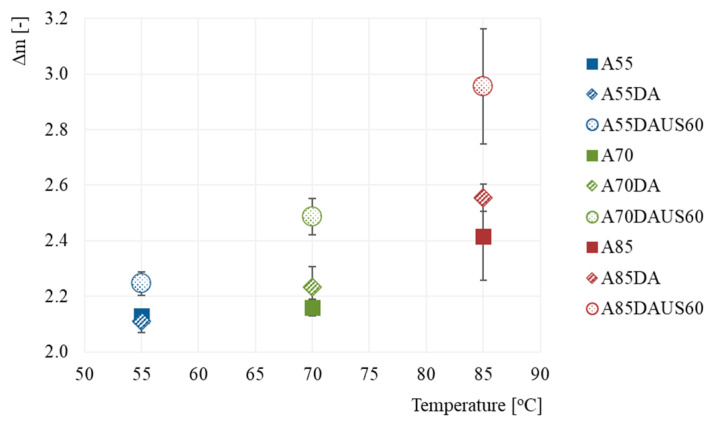
Relative mass gain (Δm) of the dried apples obtained by the convective method with non-dehumidified air, dehumidified air, and dehumidified air with ultrasound pretreatment.

The relative mass gain (Δm) of dried apples during rehydration are presented in Figure 5. All the results ranged from 2.1 to 3.0. As the temperature of the drying air rises, the relative mass gain of the product was increasing after 30 min of rehydration. Despite the lack of significant differences (*p* > 0.05), the use of dehumidified air of 70 and 85 °C increased the relative mass gain of the dried products, but when the temperature of 55 °C was set, this parameter was slightly lower than in the case of drying without dehumidified air. The application of ultrasound as a treatment prior to drying at each of the set temperatures led to obtain apples characterized by the highest mass gain. However, only in the case of 85 °C the impact of ultrasound was statistically different than for others variants of drying. Ultrasonic waves may have prevented the formation of a burned layer on the surface of the samples, which could have been a barrier to mass transport in the rehydration process, and thus increased the relative mass gain of the products [50]. This prevention could be a consequence of faster water migration from inner part of the material to its external layers. The higher rehydration ratio after ultrasound was also reported by Dadan and Nowacka [48], in the case of carrots when the treatment was carried out in ethanol solution. It was explained by the higher porosity of the material after sonication due to damage of the internal structure. It seems that samples obtained with non-dehumidified air, especially with increasing air temperature, were characterized by a higher hygroscopicity than those obtained with an air of a decreased humidity. Whereas, the materials obtained with dehumidified air, parrticularly when ultrasound was used, exhibited better rehydration ability.

**Figure 6:**
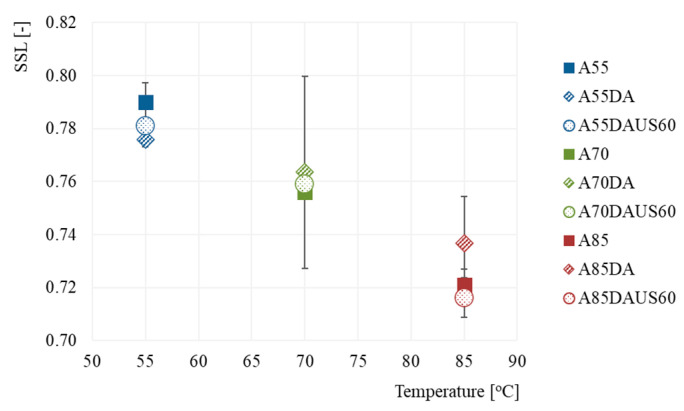
Relative dry matter content (SSL) in the dried apples obtained by the convective method with non-dehumidified air, dehumidified air, and dehumidified air with ultrasound pretreatment.

Figure 6 shows the relative dry matter content (SSL) in the dried apples after 30 min of rehydration, which ranged from 0.72 to 0.79, indicating that 21–28% of the dry matter’s components leaked from the tissue to water during rehydration. A decrease of the relative dry matter content (increased loss of dry matter) was linked to increased temperature during drying, irrespective of the other treatment conditions. Despite the lack of significant differences (*p* > 0.05), when compared with non-dehumidified air, dehumidified air at the temperature of 70 and 85 °C led to dried apples. This was characterized by a higher relative dry matter content after rehydration but drying at 55 °C resulted in the opposite behavior. The application of ultrasound treatment prior to convective drying contributed to a lack of statistically relevant differences in SSL. The materials dried with dehumidified air at 85 °C previously, subjected to ultrasound treatment, were characterized by the lowest relative dry matter content (Figure 6) and by the highest relative mass gain after rehydration (Figure 5). It can, thus, be concluded that the diffusion of water-soluble components from the product, their leakage into the environment, and water absorption were the most effective [49].

### 3.5. Color

The color parameters of dried apples are summarized in Table 2. The lightness (L*) ranged from 69.6 to 82.8. In comparison to the non-dehumidified air, the use of dehumidified air at each of the set temperature values (55, 70 and 85 °C) brightened the dried products. However, the relationship was not statistically relevant (*p* > 0.05). The samples dried with dehumidified air with preliminary ultrasound treatment were darker, in comparison to the products untreated prior to the same drying method. In the statistical terms, it was significant for temperature of 55 and 85 °C. The lowest L* value was noticed for the product pretreated by ultrasound and dried at 55°C. Darkening of apples obtained by sonication followed by drying, when compared with products untreated prior to drying at the same conditions, was also observed by Fijałkowska et al. [39]. On the contrary, Fijałkowska et al. [41] observed that sonication prior to convective drying caused the brightest color of dried apples among all others processing parameters. It is probably related to the processing parameters, not sonication itself.

The values of the a* color parameter of the dried apples ranged from −2.3 to +2.4. Rising the air temperature increased the a* values of the products dried with dehumidified air. The samples after drying at 55 °C were characterized by a positive value of a* (higher greenness) when the dehumidified air was used, whereas a negative value of a* was noted for non-dehumidified air. An opposite relationship was observed when the temperature rose to 70 °C, however, the difference was not statistically significant (*p* > 0.05). Therefore, at 85 °C both types of air contributed to obtained apples of a higher share of red color. However, with non-dehumidified air, “the concentration” of this color was significantly greater. The sample sonicated prior to drying with dehumidified air at 55 °C was less green than a sample untreated by ultrasonic waves at the same drying conditions. Among the apples dried at 70 and 85 °C, the application of ultrasound prior to drying with dehumidified air contributed to the highest share of the green color. A combination of ultrasound pretreatment with the drying with low-temperature dehumidified air, by accelerating the removal of water from the product, may have reduced the enzymatic and nonenzymatic browning reactions taking place during the drying. Fijałkowska et al. [41] observed that sonication prior to convective drying resulted in a higher greenness of dried apples than in other cases.

The dried apples were characterized by b* color parameter in the range of 25.9–33.6, which indicates a high share of yellow color. Generally, the yellowness was less dependent on the processing conditions than others color indicators. The dried apples were characterized by statistically stable b* parameter. Despite the lack of significant differences in comparison to non-dehumidified air, the use of dehumidified air at the temperature of 55 and 85 °C resulted in a decrease of b* parameter. However, for 70 °C, the yellowness of the apples dried with the air of lower humidity was greater. In contrast, the use of ultrasound treatment prior to drying of apples with dehumidified air at 55 and 70 °C resulted in a lower concentration of the yellow color in samples. An opposite relationship was observed when 85 °C was set. However, it was statistically irrelevant (*p* > 0.05). Similar results were reported by Fijałkowska et al. [41], who observed no significant differences between the b* color parameter of convectively dried apples, with or without preliminary ultrasound treatment. Therefore, the ultrasound did not change the yellowness of dried color in a statistical terms.

The browning index (*BI*) of fresh apple was equal to 36.4 (Table 2). In the case of dried apples it ranged between 38.9–57.5, indicating that drying due to the influence of oxygen and a higher temperature, contributed to the occurrence of enzymatic and nonenzymatic browning reactions during this process. As air temperature increased, the *BI* of the samples subjected to ultrasound treatment prior to drying with dehumidified air slightly decreased. However, the relationship was not significant (*p* > 0.05). In comparison to non-dehumidified air, the use of dehumidified air at a temperature of 55 and 85 °C significantly reduced the *BI* of the dried apples. However, when the temperature reached 70 °C, statistically similar results were obtained, irrespectively of the processing conditions. Furthermore, the application of ultrasound treatment did not change significantly the *BI* in comparison to drying with dehumidified air. The value of the *BI* – most similar to the raw apple–was calculated for the product dried with dehumidified air at 85 °C (*BI* = 38.9), which confirms the positive effect of a shorter drying time.

All the results of the total color difference between dried and raw apples (*ΔE*) ranged from 3.3 to 9.9. The product with the most similar color to the raw apple was dried with dehumidified air at 85 °C, which proves the significant influence of the drying time, that is, the action of oxygen on changes in the color of apple tissue. The use of dehumidified air of 55 and 85 °C gave products more similar in color to a fresh apple than drying with non-dehumidified air at the same temperature. As the temperature reached 70 °C, the use of non-dehumidified air produced a dried apple with a lower total color difference than drying with dehumidified air at the same temperature. However, the application of ultrasound treatment prior to drying led to an inverse relationship; a smaller total color difference was observed in a sample obtained by drying with dehumidified air at 70 °C, and a greater difference was observed after drying at 55 and 85 °C. Kowalski et al. [51] reported that ultrasound-assisted convective drying of apples at 40 and 50 °C contributed to an increase in the total color difference in comparison to drying unassisted by ultrasound. The values of this parameter were calculated in relation to the raw product, and in the case of all dried apples, varied between 13.5 and 19.5. This may be explained by the higher temperatures on the surfaces of the dried samples during this type of drying. Moreover, ultrasound contributing to the destruction of the thin layer of moisture on the exterior of the product by its atomization, may have increased the exposure of the product to hot air [45].

### 3.6. Total Phenolic Content and Antioxidant Activity

The polyphenols content in fresh apple amounted to 911.8 mg allic acid/100 g d.m. (dry matter). Table 3 summarizes the total phenolic content in dried apples, which varied between 628.5 and 988.9 mg/100 g d.m. The drying with dehumidified air at 55 °C did not impact on the polyphenols when compared with non-dehumidified air at the same temperature. Whereas, when 70 °C was set, a significantly higher content was noticed (by 19–27%). However, with an air temperature of 85 °C, a significantly higher phenolic content was noted in apple dried with non-dehumidified air. The application of ultrasound prior to drying with dehumidified air did not cause any significant changes in the polyphenol content. Despite the lack of statistically significant differences (*p* > 0.05), the ultrasonic treatment coupled with drying at 70 and 85 °C led to a slight increase of the polyphenols content, while in the case of the lowest temperature of 55 °C, a slight reduction was observed. The highest polyphenol content was reported in the product dried with dehumidified air at 70 °C preceded by ultrasound treatment. The higher polyphenol content in some of the dried apples (A70DA, A70DAUS60) than in the fresh apples could have been due to the presence of other antioxidant substances (e.g., browning products) produced during drying, which reacted with the Folin-Ciocalteu reagent [52]. Moreover, subjecting the apples to ultrasound pretreatment reduced the time of drying with dehumidified air, so the exposure of the product to hot air and oxygen was also reduced, which probably contributed to a greater retention of polyphenols. Additionally, tissue damage caused by ultrasound and a high temperature may lead to increased extractivity of polyphenols [53]. Rodrigues et al. [54] observed that both convective drying and ultrasound-assisted drying did not impact significantly the content of polyphenols in dried apples when compared with the raw product. Once the temperature of the drying air were increased from 45 to 60 °C, a significant reduction of polyphenols in the samples obtained with convective drying was observed. However, the application of ultrasound significantly improved their retention.

The concentration of extract necessary to scavenge the 50% of DPPH radical was equaled to 0.707 mg d.m./mL for fresh apple. In the case of dried apples, the EC_50_ ranged from 0.835 to 1.404 mg d.m./mL. As the temperature of the drying air increased, the antioxidant activity of the dried apples obtained by drying with non-dehumidified air decreased. Considering the degree of humidity of the drying air, it may be concluded that the use of dehumidified air at a temperature of 70 °C led to a sample with a significantly higher antioxidant activity than one dried with non-dehumidified air at the same temperature. Despite the lack of significant differences, the use of dehumidified air at a temperature of 55 and 85 °C resulted in a dried product with a higher, and lower antioxidant activity, respectively, than drying with non-dehumidified air at the same temperatures. The application of ultrasound prior to drying with dehumidified air did not differentiate significantly the obtained antioxidant activity. However, it was noticeable that when ultrasound coupled with drying with dehumidified air at 55 and 85 °C were applied, a slight increase in their antioxidant activity was noted. Therefore, the highest antioxidant activity and, at the same time, the highest phenolic content was noticed in the case of drying with dehumidified air at a temperature of 70 °C preceded by ultrasound treatment. It shows that there are some “optimal” parameters (from phenolics retention point of view) that compromise both long time of treatment and thus long exposure for oxygen with the impact of high temperature.

## 4. Conclusions

Ultrasound pretreatment and dehumidified air can reduce drying time by 11–15% when compared to the control process performed using untreated material and ambient air. Samples processed at 50 and 70 °C using ultrasound and dehumidified air exhibited higher retention of phenolics (7–27%) and better antioxidant activity (13–32%). These results are most likely related to the shorter drying time and the exposition on oxygen from air and elevated temperatures. Moreover, such treated materials exhibited better reconstitution properties, which most probably was a consequence of the changes in the microstructure of the samples provoked by sonication. However, this requires further investigation in order to confirm such explanation. Since the effect of ultrasonic waves and dehumidified air on process kinetics and studied properties was most visible at lower drying air temperatures, it seems justifiable to conduct further research with a range of lower process temperatures. Such behavior may be caused by the fact, that at high temperature, the effects of sonication and dehumidified air are covered by very intensive water evaporation.

## Figures and Tables

**Table 1:** Dry matter content and water activity of dried apples obtained by the convective method with non-dehumidified air (A55, A70, A85), dehumidified air (A55DA, A70DA, A85DA), and dehumidified air with ultrasound pretreatment (A55DAUS60, A70DAUS60, A85DAUS60); the same letters (a, b, etc.) in the columns represent homogeneous groups in statistical terms (α = 0.05).

Sample	Dry Matter Content [%]	Water Activity
A55	92.95 ± 0.18 ^c^	0.266 ± 0.011 ^d^
A55DA	91.54 ± 0.07 ^b^	0.307 ± 0.002 ^e^
A55DAUS60	91.09 ± 0.06 ^a^	0.339 ± 0.006 ^f^
A70	95.37 ± 0.18 ^e^	0.233 ± 0.000 ^c^
A70DA	93.10 ± 0.06 ^c^	0.277 ± 0.006 ^d^
A70DAUS60	94.85 ± 0.12 ^d^	0.223 ± 0.004 ^bc^
A85	98.88 ± 0.02 ^g^	0.200 ± 0.001 ^ab^
A85DA	96.74 ± 0.00 ^f^	0.195 ± 0.001 ^a^
A85DAUS60	96.56 ± 0.06 ^f^	0.222 ± 0.012 ^bc^

**Table 2:** The color of the dried apples obtained by the convective method with non-dehumidified air (A55, A70, A85), dehumidified air (A55DA, A70DA, A85DA), and dehumidified air with ultrasound pretreatment (A55DAUS60, A70DAUS60, A85DAUS60); the same letters (a, b, etc.) in the columns represent homogeneous groups in statistical terms (α = 0.05).

Sample	L*	a*	b*	*BI*	*ΔE*
A55	76.7 ± 3.3 ^abc^	1.5 ± 1.9 ^cd^	33.6 ± 4.2 ^b^	57.5 ± 11.3 ^d^	9.9
A55DA	82.8 ± 2.5 ^c^	−2.3 ± 0.3 ^a^	29.5 ± 3.1 ^ab^	40.8 ± 4.9 ^ab^	8.6
A55DAUS60	69.6 ± 8.5 ^a^	−0.4 ± 1.0 ^b^	28.1 ± 4.2 ^ab^	49.8 ± 6.5 ^bcd^	8.8
A70	78.0 ± 3.8 ^bc^	−0.2 ± 1.2 ^b^	29.0 ± 4.9 ^ab^	45.1 ± 9.1 ^abc^	5.4
A70DA	78.6 ± 4.0 ^c^	0.0 ± 0.9 ^bc^	32.4 ± 4.3 ^b^	51.7 ± 8.8 ^cd^	8.9
A70DAUS60	76.8 ± 3.4 ^abc^	−0.2 ± 0.6 ^b^	29.3 ± 2.3 ^ab^	46.4 ± 3.8 ^abc^	5.7
A85	75.5 ± 3.4 ^abc^	2.4 ± 0.3 ^d^	29.3 ± 2.6 ^ab^	50.3 ± 4.7 ^bcd^	6.1
A85DA	79.7 ± 2.5 ^c^	0.6 ± 1.8 ^bc^	25.9 ± 2.1 ^a^	38.9 ± 4.2 ^a^	3.3
A85DAUS60	70.5 ± 10.4 ^ab^	−0.8 ± 0.8 ^ab^	26.2 ± 5.9 ^a^	44.1 ± 7.7 ^abc^	7.3

**Table 3:** Total phenolic content and antioxidant activity of dried apples obtained by the convective method with non-dehumidified air (A55, A70, A85), dehumidified air (A55DA, A70DA, A85DA), and dehumidified air with ultrasound pretreatment (A55DAUS60, A70DAUS60, A85DAUS60); the same letters (a, b, etc.) in the columns represent homogeneous groups in statistical terms (α = 0.05).

Sample	Total Phenolic Content [mg gallic acid/100 g d.m.]	EC_50_ DPPH [mg d.m./mL]
A55	779.86 ± 26.41 ^bc^	1.150 ± 0.018 ^cd^
A55DA	841.59 ± 51.74 ^cd^	1.065 ± 0.039 ^bc^
A55DAUS60	837.61 ± 5.65 ^cd^	0.996 ± 0.028 ^b^
A70	778.40 ± 8.45 ^bc^	1.230 ± 0.003 ^de^
A70DA	922.43 ± 12.61 ^de^	0.852 ± 0.076 ^a^
A70DAUS60	988.91 ± 61.07 ^e^	0.835 ± 0.024 ^a^
A85	795.30 ± 9.12 ^bcd^	1.352 ± 0.029 ^ef^
A85DA	628.46 ± 45.46 ^a^	1.404 ± 0.024 ^f^
A85DAUS60	698.70 ± 10.37 ^ab^	1.319 ± 0.011 ^ef^

## Data Availability

The data presented in this study are available on request from the corresponding author.
